# RETRACTION: Surgical Site Wound Infection, and Other Postoperative Problems After Coronary Artery Bypass Grafting in Subjects with Chronic Obstructive Pulmonary Disease: A Meta‐Analysis

**DOI:** 10.1111/iwj.70462

**Published:** 2025-04-02

## Abstract

**RETRACTION:** GaoJ. , WangH. , LiuX. , SongX. , and ZhongX. , “Surgical Site Wound Infection, and Other Postoperative Problems After Coronary Artery Bypass Grafting in Subjects with Chronic Obstructive Pulmonary Disease: A Meta‐Analysis,” International Wound Journal 20, no. 2 (2023): 302–312, 10.1111/iwj.13877.35801278 PMC9885461

The above article, published online on 07 July 2022, in Wiley Online Library (http://onlinelibrary.wiley.com/), has been retracted by agreement between the journal Editor in Chief, Professor Keith Harding; and John Wiley & Sons Ltd. Following an investigation by the publisher, all parties have concluded that this article was accepted solely on the basis of a compromised peer review process. The editors have therefore decided to retract the article. The authors did not respond to our notice regarding the retraction.

**RETRACTION:** J. Gao , H. Wang , X. Liu , X. Song , and X. Zhong , “Surgical Site Wound Infection, and Other Postoperative Problems After Coronary Artery Bypass Grafting in Subjects with Chronic Obstructive Pulmonary Disease: A Meta‐Analysis,” International Wound Journal 20, no. 2 (2023): 302–312, 10.1111/iwj.13877.35801278 PMC9885461

The above article, published online on 07 July 2022, in Wiley Online Library (http://onlinelibrary.wiley.com/), has been retracted by agreement between the journal Editor in Chief, Professor Keith Harding; and John Wiley & Sons Ltd. Following an investigation by the publisher, all parties have concluded that this article was accepted solely on the basis of a compromised peer review process. The editors have therefore decided to retract the article. The authors did not respond to our notice regarding the retraction.

